# Innovative Smart Materials in Restorative Dentistry

**DOI:** 10.3390/jfb16090318

**Published:** 2025-08-30

**Authors:** Roxana Ionela Vasluianu, Livia Bobu, Iulian-Costin Lupu, Magda Antohe, Bogdan Petru Bulancea, Antonia Moldovanu, Ovidiu Stamatin, Catalina Cioloca Holban, Ana Maria Dima

**Affiliations:** 1 Department of Prosthodontics, Faculty of Dental Medicine, “Grigore T. Popa” University of Medicine and Pharmacy, 700115 Iasi, Romania; 2 Department of Surgicals, Faculty of Dental Medicine, “Grigore T. Popa” University of Medicine and Pharmacy, 700115 Iasi, Romania; livia.bobu@umfiasi.ro; 3 Department of Dental Prosthesis Technology, Faculty of Dental Medicine, “Grigore T. Popa” University of Medicine and Pharmacy, 700115 Iasi, Romania; 4 Department of Odontology, Periodontology and Fixed Prosthesis, Faculty of Dental Medicine, “Grigore T. Popa” University of Medicine and Pharmacy, 700115 Iasi, Romania; 5 Independent Researcher, 700115 Iasi, Romania

**Keywords:** smart materials, bioactive, antibacterial, restorative dentistry, stimuli-responsive, pH-responsive, bioactive glass, silver nanoparticle, chitosan, oral medicine

## Abstract

The growing challenge of biofilm-associated infections in dentistry necessitates advanced solutions. This review highlights the potential of smart bioactive and antibacterial materials—bioactive glass ceramics (BGCs), silver nanoparticle (AgNP)-doped polymers, and pH-responsive chitosan coatings—in transforming restorative dentistry. BGCs reduce biofilms by >90% while promoting bone integration. AgNP-polymers effectively combat *S. mutans* and *C. albicans* but require controlled dosing (<0.3 wt% in PMMA) to avoid cytotoxicity. Chitosan coatings enable pH-triggered drug release, disrupting acidic biofilms. Emerging innovations like quaternary ammonium compounds, graphene oxide hybrids, and 4D-printed hydrogels offer on-demand antimicrobial and regenerative functions. However, clinical translation depends on addressing cytotoxicity, standardizing antibiofilm testing (≥3-log CFU/mL reduction), and ensuring long-term efficacy. These smart materials pave the way for self-defending restorations, merging infection control with tissue regeneration. Future advancements may integrate AI-driven design for multifunctional, immunomodulatory dental solutions.

## 1. Introduction

The persistent threat of microbial biofilms remains a formidable challenge in dentistry, driving prosthetic failure and compromising long-term clinical outcomes [1,2]. The oral cavity, a dynamic and microbiologically complex environment, harbors pathogens such as *Candida albicans*, *Streptococcus mutans*, *Porphyromonas gingivalis*, and more, which readily adhere to teeth and dentures surfaces, forming resilient polymicrobial consortia [3,4]. These biofilms act as reservoirs for chronic infection, evading host defenses and conventional antimicrobial therapies through protective extracellular matrices and intrinsic resistance mechanisms [5,6,7]. Clinically, this manifests as biofilm-mediated pathologies, including denture stomatitis in removable prosthodontics and mucositis in implantology, conditions that frequently progress to peri-implantitis, a leading cause of implant failure characterized by inflammatory bone destruction [8,9,10,11].

Conventional prosthodontic materials, such as polymethyl methacrylate (PMMA) for denture bases, provide essential mechanical functionality but lack inherent antimicrobial properties, rendering them vulnerable to microbial colonization [12,13,14,15]. Current adjunctive strategies—topical antifungals, systemic antibiotics, and mechanical cleaning—are often inconsistent, prone to resistance, and fail to provide sustained protection [16,17,18,19]. The rise of antibiotic-resistant strains further exacerbates these limitations, underscoring the urgent need for innovative antimicrobial solutions that transcend traditional approaches [20,21].

In response, the field has witnessed the emergence of smart bioactive and antibacterial materials engineered systems designed to dynamically counteract microbial adhesion, release antimicrobial agents in response to pathological triggers (e.g., pH shifts, enzymatic activity), and promote tissue integration [22,23,24]. These materials represent a fundamental shift from passive infection resistance to active, stimuli-responsive defense mechanisms [25,26,27]. Among the most promising innovations are bioactive glasses (BG), which release antimicrobial ions (e.g., Ag^+^, Zn^2+^, Sr^2+^) while stimulating bone regeneration; nanoparticle-enhanced polymers, incorporating metallic (Ag, Cu) or organic (chitosan, quaternary ammonium compounds) nanoparticles for sustained antimicrobial release; and pH-responsive coatings, which modulate drug delivery in acidic microenvironments induced by bacterial glycolysis or inflammation [28,29,30,31,32,33]. By integrating stimulus-responsive functionality, these advanced materials achieve targeted antibiofilm action without compromising biocompatibility, offering a sustainable solution to infection control in oral rehabilitation [34,35,36].

Given these advancements, this review synthesizes the latest developments in smart bioactive glass, silver nanoparticle polymers, and chitosan-based pH-responsive coatings for prosthodontics, holistically reviewing their mechanisms, biological interactions, and clinical applicability. It also examines how these materials address the limitations of conventional prostheses by providing dynamic, on-demand antimicrobial activity while supporting tissue integration—a dual functionality indispensable for long-term prosthetic success. By contextualizing these innovations within the broader challenges of biofilm resilience and antibiotic resistance, this review aims to guide future research and clinical translation, ultimately advancing infection control strategies in modern restorative dentistry.

## 2. Key Smart Materials in Restorative Dentistry

The development of smart bioactive and antibacterial materials has revolutionized prosthodontics by integrating antimicrobial, stimuli-responsive, and tissue-regenerative functionalities into dental prostheses and implants [37,38,39,40]. These materials are designed to combat microbial infections while promoting osseointegration and long-term prosthetic success [41,42,43]. Current section explores the most advanced materials, their biological interactions, formulations, and clinical impact in modern prosthodontics.

### 2.1. Bioactive Glass Ceramics (BGCs): Antimicrobial and Osteoconductive Dual Functionality

Bioactive glass (BG) has gained prominence in prosthodontics due to its dual capacity to release antimicrobial ions (e.g., Ag^+^, Zn^2+^) and stimulate hydroxyapatite formation, enhancing bone-implant integration [44,45,46]. When incorporated into PMMA dentures or implant coatings, BG disrupts bacterial cell membranes via ion exchange while promoting remineralization of adjacent tissues [47,48,49]. Recent formulations also exhibit fungicidal activity against Candida species, addressing a serious constraint of conventional denture materials [50,51].

While bioactive glass (BG) is an entirely amorphous (non-crystalline) material, bioactive glass-ceramics (BGCs) consist of at least one crystalline phase embedded within a glassy matrix. The incorporation of crystalline phases enhances the mechanical properties of BGCs, often resulting in greater strength and fracture toughness compared to BG. BGCs epitomized by formulations like S53P4, F18, and Advanced Modifications, represent a breakthrough in biomaterials for prosthodontics and implantology [52,53,54,55,56,57]. These remarkable materials transcend passive roles, actively orchestrating a symphony of biological responses through the controlled release of therapeutic ions (Ca^2+^, Zn^2+^, Cu^2+^, Ag^+^, and Sr^2+^) [58,59,60]. Their core innovation lies in dual functionality: simultaneously combating microbial threats while fostering robust osseointegration and tissue regeneration [61,62,63,64,65,66,67]. This synergy addresses significant shortcomings of traditional materials, positioning BGCs as cornerstone elements in next-generation dental restorations [68,69,70].

*Biological Interactions*

Bioactive glass ceramics (BGCs) achieve their therapeutic efficacy through three primary, interrelated mechanisms: antimicrobial ion release, osteostimulation, and angiogenic promotion [71,72,73]. These mechanisms function synergistically to ensure prosthetic success by concurrently addressing the serious challenges of infection control and tissue regeneration [74,75]. The fundamental therapeutic power of BGCs originates from their controlled dissolution kinetics that releases a precisely engineered cascade of therapeutic ions [76,77].

The antimicrobial defenses are multifaceted. Firstly, ion exchange antagonism occurs as alkaline ions, such as Ca^2+^ and SiO_4_^4−^, elevate the local pH, creating a bacteriostatic and fungistatic alkaline environment detrimental to acidogenic pathogens like *Streptococcus mutans* and *Candida albicans* [63,78,79,80]. Secondly, divalent cations, notably Zn^2+^ (in Zn4 BGCs) and Ag^+^, directly target microbial membranes, inducing depolarization, compromising structural integrity, and triggering lethal reactive oxygen species (ROS) generation [81,82,83,84]. This membrane disruption mechanism devastates biofilms of key pathogens including *Porphyromonas gingivalis*, *Aggregatibacter actinomycetemcomitans*, and *S. mutans*, achieving greater than 90% reductions in viability [61,85,86]. This antimicrobial potency is further enhanced by sustained nanoscale delivery systems. Advanced structures, such as Ag^+^-doped mesoporous films (80SiO_2_-15CaO-5P_2_O_5_), provide controlled, sustained release of Ag^+^, ensuring prolonged membrane disruption while optimized Ca/P ratios simultaneously facilitate concurrent hydroxyapatite nucleation [87,88]. Ag-hydroxyapatite (Ag-HA) nanocoatings exemplify this dual functionality, achieving near-total bacterial mortality (100% planktonic, 97.5% biofilm reduction) coupled with exceptional stability demonstrated by less than 0.07% dissolution [89,90].

Concurrently, BGC dissolution drives osteostimulation and angiogenesis [91,92]. The released Ca^2+^ and PO_4_^3−^ ions supersaturate the local environment, triggering the formation of a biologically active hydroxycarbonate apatite (HCA) layer directly on the BGC surface [93]. This HCA layer is chemically and structurally analogous to natural bone mineral, serving as an osteoconductive scaffold for osteoblast adhesion and subsequent new bone deposition. Furthermore, specific therapeutic ions act as potent molecular signals orchestrating cellular activity. Cu^2+^ contributes to the upregulation of key osteogenic transcription factors, such as RUNX2, and bone matrix proteins like osteocalcin, thereby accelerating bone matrix deposition [94]. Simultaneously, Cu^2+^ stimulates vascular endothelial growth factor (VEGF) secretion, promoting a significant 3.5-fold increase in angiogenesis, which is important for nutrient delivery and long-term implant survival [63]. Sr^2+^ mimics calcium signaling, directly enhancing osteoblast activity and proliferation while inhibiting osteoclast resorption, leading to improved bone density [95]. Beyond its established antimicrobial action, Zn^2+^ also functions as an essential co-factor for numerous enzymes involved directly in bone formation and mineralization processes (Table 1).This integrated ion-mediated cellular orchestration underpins the remarkable regenerative capacity of BGCs [96].

*Advanced Formulations*

The true power of bioactive glass ceramics (BGCs) resides fundamentally in their inherent tunability, enabling modern modifications to directly address specific clinical challenges. A significant advancement involves zirconium-modified BGCs (Zr-BGCs), developed to reconcile the need for both robust mechanical strength and sustained bioactivity in load-bearing prosthodontic applications such as implants and abutments. Recent advancements demonstrate that zirconium (Zr) incorporation significantly enhances the overall performance profile of these materials. Specifically, the integration of ZrO_2_ elevates fracture resistance by 40% compared to conventional BGCs, while preserving essential bioactive behavior [97]. Notably, Zr-BGCs maintain their characteristic ion release kinetics, which underpins sustained antimicrobial efficacy; this is exemplified by an 80% reduction in *Staphylococcus aureus* adhesion [63]. Furthermore, these materials actively promote osteogenic differentiation, offering the dual benefit of increased mechanical durability alongside enhanced osseointegration potential. This synergy between strength and bioactivity positions Zr-BGCs as highly suitable for high-stress prosthetic components.

Addressing the need for rapid vascularization and bone formation, particularly in compromised sites, has led to the development of copper-doped mesoporous bioactive glass (Cu-MBG). Applying these nanostructured mesoporous coatings, for example onto titanium implants, doped with Cu^2+^, delivers a multifaceted therapeutic effect. These coatings provide significant antibacterial action, such as a fivefold reduction in *P. gingivalis*, coupled with powerful pro-angiogenic and osteogenic signaling through controlled Cu^2+^ ion release [61,98]. This coordinated action significantly accelerates the establishment of a viable bone-implant interface during the delicate early stages of osseointegration.

In conclusion, bioactive glass ceramics like S53P4, F18, and their advanced derivatives, including Ag-doped formulations, Cu-MBG, Zr-BGCs, and Nb-modified compositions, represent a sophisticated evolution in biomaterial science. They transcend the role of mere structural replacements, functioning instead as dynamic therapeutic platforms. By harnessing the precise ionic language of controlled release—Ca^2+^ and PO_4_^3−^ for bone matrix construction, Ag^+^ and Zn^2+^ acting as microbial sentinels, and Cu^2+^ and Sr^2+^ directing cellular processes like conductors—these materials actively design their biological environment. This exquisite integration of antimicrobial efficacy, osteogenic potential, angiogenic induction, and tunable mechanical properties, robustly supported by scientific evidence, firmly establishes BGCs as indispensable, multifaceted tools for achieving long-term success in the complex domains of restorative and regenerative dentistry.

### 2.2. Silver Nanoparticle-Doped Polymers: Prolonged Antimicrobial Defense

Nanotechnology has revolutionized prosthodontic polymers by embedding them with antimicrobial nanoparticles (e.g., silver, copper, or graphene oxide), which provide sustained release and broad-spectrum antibacterial effects [99]. For instance, silver nanoparticles (AgNPs) integrated into PMMA demonstrate potent activity against *Streptococcus mutans* and *Staphylococcus aureus*, reducing biofilm formation by up to 90% [100,101,102,103].

*Biological Interactions*

**Figure 1:**
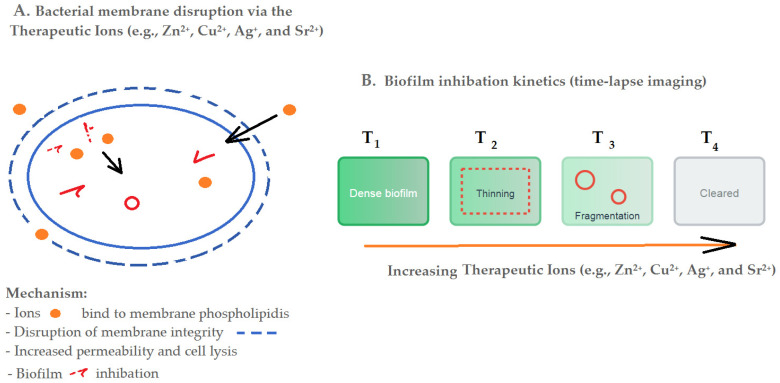
Generalized Model of Ion Release Mechanisms ((**A**). Bacterial membrane disruption via therapeutic ions; (**B**). Biofilm inhibition kinetics).

The antimicrobial efficacy of AgNPs stems from multiple mechanisms. Silver ions (Ag^+^) released from nanoparticles induce bacterial membrane lysis and DNA damage, while ROS generation further disrupts microbial integrity [104,105,106,107]. A broad conceptual summary of ion release mechanisms is depicted in Figure 1.

For instance, AgNPs immobilized on UiO-66 frameworks, a metal–organic framework, prevent particle aggregation and enhance ROS-mediated membrane rupture [108,109]. Similarly, β-AgVO_3_-decorated adhesives at low concentrations (1–10 wt%) demonstrate potent activity, eradicating *Streptococcus mutans* at 1% loading and reducing *Candida albicans* viability by 4-log [110].

A major issue in AgNP applications is balancing antimicrobial efficacy with biocompatibility, necessitating controlled release strategies for toxicity mitigation. Uncontrolled Ag^+^ release can lead to cytotoxicity, necessitating engineered delivery systems. For example, AgNP-anchored TiO_2_ nanotubes with a nano-hydroxyapatite (nHA) top-layer exhibit moderated ion dissolution (3.27 μg/L over 24 h), reducing biofilm formation by 80% without burst release [111]. Similarly, silica-EDTA nanocomposites and chitosan-conjugated AgNPs (Ag-Chi) have been shown to inhibit *S. mutans* and *Porphyromonas gingivalis* adhesion while minimizing cytotoxic effects [79,112].

*Synthesis & Optimization*

**Figure 2:**
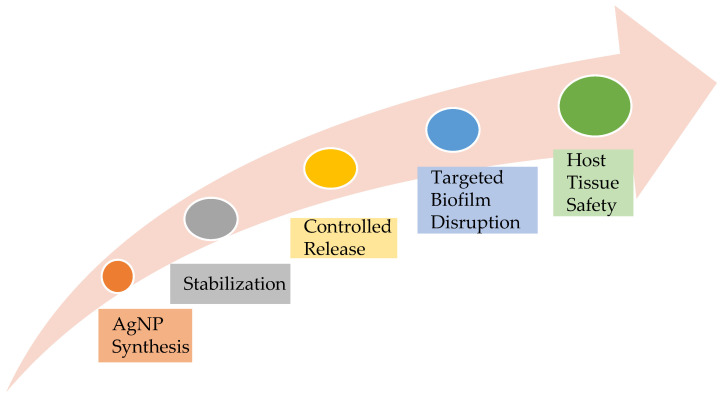
AgNP—from Synthesis to Performance.

The performance of AgNP-doped polymers hinges on precise synthesis techniques that govern particle size, distribution, and release kinetics, significantly influencing AgNP stability and performance. The convergence of these strategies enables next-generation antimicrobial polymers (Figure 2).

**Figure 3:**
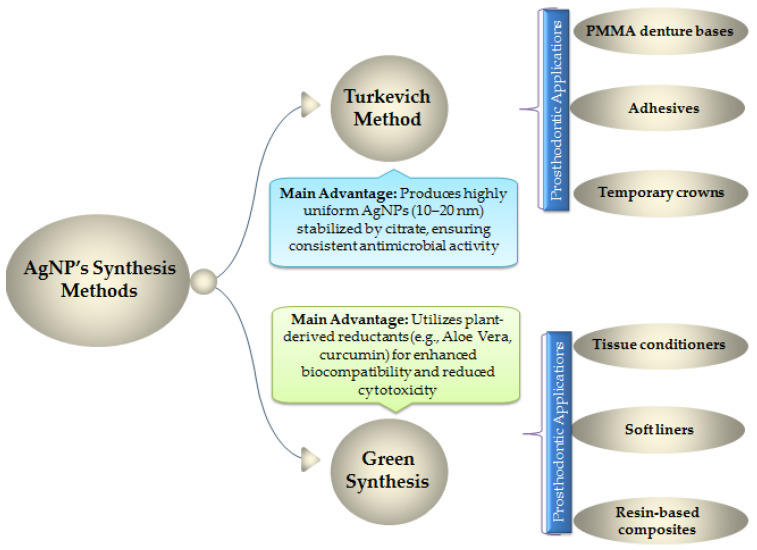
Comparison of Silver Nanoparticle Synthesis Methods: Turkevich Method vs Green Synthesis Approaches.

While AgNP synthesis can be achieved through physical, chemical, and biological approaches, two primary techniques are most prominent in contemporary studies: the Turkevich Method, and the Green Synthesis [113,114]. The Turkevich method yields citrate-capped AgNPs with uniform size distribution, while green synthesis using plant-derived reductants (e.g., Aloe vera, Curcuma longa) enhances biocompatibility without compromising antimicrobial (Figure 3).

The Turkevich (citrate reduction) method is a well-established chemical synthesis approach in which citrate ions serve a dual role as both the reducing agent for silver ions (Ag^+^) and the stabilizing ligand for the resulting nanoparticles. This method produces colloidal silver nanoparticles (AgNPs) with high monodispersity, typically ranging between 10–20 nm in diameter. The citrate capping mechanism provides electrostatic stabilization, preventing nanoparticle aggregation and ensuring batch-to-batch reproducibility. This uniformity is necessary for achieving consistent and potent antimicrobial activity, making Turkevich-synthesized AgNPs particularly valuable in restorative dentistry. Their reliable antimicrobial properties facilitate incorporation into polymethyl methacrylate (PMMA) acrylic resin for denture bases, denture adhesives, and provisional crown materials, effectively reducing microbial colonization and biofilm formation.

In contrast, phytogenic (green) synthesis leverages plant-derived extracts or purified phytochemicals (Aloe Vera leaves, curcumin, or other), as reducing and capping agents for AgNP formation. The complex mixture of biomolecules (e.g., polyphenols, flavonoids, terpenoids, and proteins) within these extracts mediates the reduction of Ag^+^ ions while simultaneously stabilizing the resulting nanoparticles. A key advantage of this approach is the enhanced biocompatibility and reduced cytotoxicity conferred by the biological capping agents, compared to nanoparticles synthesized using harsher chemical methods. Additionally, green synthesis is considered more environmentally sustainable. Given their favorable biocompatibility profile, phytogenically synthesized AgNPs are particularly well-suited for applications involving prolonged mucosal contact, such as tissue conditioners and resilient soft denture liners. They are also incorporated into resin-based composite materials for dental restorations, where minimizing tissue irritation is essential.

Thus, while the Turkevich method offers precision and reproducibility for antimicrobial applications, green synthesis provides a biocompatible and eco-friendly alternative, expanding the potential of AgNPs in diverse prosthodontic applications.

*Material-Specific Enhancement*

Incorporating AgNPs into dental polymers improves both antimicrobial and mechanical properties. Polymethyl methacrylate (PMMA) doped with Novaron (Ag/ZrPO_4_) or T-ZnO reduces biofilm colony-forming units (CFUs) by >95% while enhancing flexural strength [115]. Likewise, heat-cured resins containing 10% AgVO_3_ achieve >99% reduction in multi-species biofilms, though *C. albicans* resistance has been noted in some formulations [116]. Ion-implanted AgNPs (58 nm) have also demonstrated efficacy, reducing *Staphylococcus aureus* colonization by 64.6% [117].

Recent innovations, such as metal–organic framework (MOF)-stabilized AgNPs (e.g., UiO-66/AgNP nanocomposites), have further refined controlled release kinetics, preventing nanoparticle aggregation while minimizing cytotoxic risks [109]. These advancements ensure that antimicrobial potency is maintained without compromising host cell viability, a fundamental equilibrium in long-term prosthodontic applications.

The integration of AgNPs into prosthodontic materials represents a significant advancement in preventing biofilm-associated infections. By leveraging controlled release mechanisms, advanced immobilization techniques, and biocompatible synthesis approaches, researchers have optimized AgNP-doped polymers for clinical use. Future developments may focus on overcoming microbial resistance (e.g., *C. albicans* adaptation) and refining dose–response profiles to maximize therapeutic efficacy while ensuring patient safety.

### 2.3. Chitosan-Based pH-Responsive Coatings

Chitosan (CS), a natural cationic polysaccharide derived from the deacetylation of chitin, stands as a versatile biopolymer with inherent antimicrobial, mucoadhesive, and pH-responsive properties [118,119]. This unique combination, particularly its ability to undergo reversible protonation/deprotonation of primary amine groups across physiological pH ranges, underpins its utility in designing “smart” coatings. These coatings exhibit tailored responses, especially within acidic microenvironments characteristic of bacterial biofilms, carious lesions, or infected peri-implant pockets. The inherent biocompatibility and biodegradability of chitosan further enhance its suitability for biomedical and dental applications, such as modifying glass-ionomer cements (GICs). Chitosan-modified GICs have demonstrated enhanced antibacterial properties against *Streptococcus mutans*, a key bacterium in tooth decay, while also improving mechanical strength and fluoride release. These properties make chitosan a promising additive for dental materials, including denture liners, implant coatings, and caries-preventive GICs [120,121,122]. Its fundamental characteristic is the shift from a neutral/insoluble state at physiological pH to a soluble, positively charged polyelectrolyte under acidic conditions, driving key functional mechanisms.

*Biological Interactions*

The efficacy of chitosan-based pH-responsive coatings primarily stems from two interconnected mechanisms activated in acidic milieus (typically pH < 5.5). Firstly, the pH-dependent structural and charge modulation is essential. Under acidic conditions, protonation of chitosan’s amine groups (-NH_3_^+^) induces polymer swelling, electrostatic repulsion, and increased solubility. This charge reversal enhances interaction with negatively charged microbial membranes and promotes biofilm penetration [123,124]. Secondly, this protonation triggers controlled therapeutic release. The swelling and structural changes disrupt the coating matrix or cleave pH-sensitive linkages (e.g., Schiff bases like chitosan-citral complexes), enabling the release of encapsulated or complexed agents such as fluoride, Zn^2+^, antibiotics (e.g., amoxicillin, minocycline, metronidazole), antifungal agents, gallium, D-arginine, or growth factors [125,126,127,128]. Advanced systems like redox/pH-triggered nanocomposites (e.g., chitosan/ethylcellulose) or sequential release platforms (e.g., pore-closed PLGA microparticles in chitosan hydrogel) offer intricate temporal control [128,129]. The released agents and the cationic chitosan itself exert potent antimicrobial/antifungal effects. Mechanisms include cationic disruption and destabilization of microbial membranes (leading to leakage), particularly effective against fungi like *C. albicans* on resins chelation of essential nutrients (e.g., Zn^2+^ chelation), and disruption of quorum sensing and bacterial adhesion [130,131,132,133].

*Diverse Formulations*

Diverse chitosan-based formulations leverage these mechanisms for targeted applications. Nanoparticles and Microparticles are prominent: Fluoride-loaded chitosan nanoparticles incorporated into GICs significantly reduce secondary caries depth (by 9%) through enhanced remineralization and localized fluoride release [122]. Aspirin and amoxicillin co-loaded microparticles utilize pH-dependent erosion for sustained dual-drug elution, achieving remarkable 99% metabolic biofilm reduction while also enhancing rBMSC adhesion [134]. Hydrogels and Films provide versatile platforms: High molecular weight chitosan gel demonstrates potent inhibition (100%, *p* < 0.001) of *C. albicans* adherence to acrylic resin [132]. Lysostaphin-chitosan hydrogels achieve substantial reductions in *S. aureus* viability (~3 Log_10_ CFU/mL) in ex vivo models [135]. Multicomponent Composites and Layer-by-Layer (LBL) Coatings enhance functionality: Zn^2+^-chelated chitosan/gelatin composites disrupt bacterial adhesion (90% reduction) and quorum sensing while accelerating human gingival fibroblast migration [62]. Quaternary ammonium chitosan/alginate LBL coatings maintain exceptional anti-biofilm efficacy (>90% reduction against Candida) even after mechanical stress, important for enduring applications [133]. Advanced Nanocomposites represent cutting-edge development: Zeolitic imidazolate frameworks (ZIF-8) loaded with naringin on TiO_2_ nanotubes, integrated within chitosan systems, enable pH-responsive Zn^2+^ release, synergistically combining antibacterial and osteogenic effects [87] (Table 2).

## 3. Emerging Smart Materials in Dentistry: Quaternary Ammonium Compounds, Graphene Oxide, and Beyond

Key material classes driving this innovation include quaternary ammonium compounds (QACs), graphene oxide (GO)-based hybrids, multifunctional hydrogels, metal-ion releasing platforms, and nanocomposites like zeolitic imidazolate frameworks (ZIF-8). Their integration into dental polymers, implant coatings, and tissue engineering scaffolds represents a transformation towards inherently bioactive prosthodontic applications, aiming to enhance longevity and reduce infection-related failures.

*Biological Interactions*

The antimicrobial efficacy of these smart materials stems from distinct, often synergistic, biological interactions. QACs, exemplified by quaternary ammonium-polyethyleneimine (QPEI) grafted onto titanium or quaternary ammonium monomers (QA-P) covalently bound to polymethyl methacrylate (PMMA), primarily exert their effects through electrostatic disruption of bacterial membranes. This interaction, driven by the cationic nature of QACs attracting the anionic bacterial surface, compromises membrane integrity leading to cell death [136,137]. Graphene oxide (GO) contributes through mechanical action, where its sharp nanosheets physically puncture bacterial membranes, while also providing mechanical reinforcement to polymers like PMMA [138]. Photocatalytic systems, such as graphene oxide/tungsten trioxide/silver bromide/silver (GO/WO_3_/AgBr/Ag) and titanium dioxide/molybdenum diselenide/chitosan (TiO_2_/MoSe_2_/chitosan), generate cytotoxic ROS under light irradiation, visible and near-infrared (NIR), respectively, causing oxidative damage to biomolecules within biofilms [139]. Metal ions, like Sr^2+^ released from strontium-functionalized titanium or Cu^2+^ from copper-nanoparticle hydrogels, disrupt microbial metabolism through bacteriostatic effects and induction of oxidative stress [140,141].

*Formulations*

The practical application of these mechanisms relies on sophisticated material formulations. Covalent surface modification is a prominent strategy, demonstrated by QPEI grafting onto titanium implant surfaces and QA-P monomers incorporated into the PMMA matrix of dentures or provisional restorations [136,137]. Double cross-linked hydrogels represent another advanced formulation; quaternized chitosan/polyacrylamide-polydopamine (QCS/PAM-PDA) hydrogels achieve exceptional antibacterial efficacy (99.99%) while maintaining robust mechanical properties (13.3 kPa tensile strength), making them suitable for soft tissue interfaces or drug delivery matrices. Nanocomposite formulations are widely employed: GO is integrated into PMMA for enhanced flexural strength and inherent antibacterial action, often combined with silver nanoparticles (GO-AgNP) for synergistic effects in denture bases. Photocatalysts are typically formulated as coatings or embedded particles, such as the GO/WO_3_/AgBr/Ag composite used for light-activated biofilm degradation. Ruthenium-based complexes, specifically shikimate-crosslinked chitosan-ruthenium(II) (Ru(II)), offer sustained antibacterial release and are explored for implant coatings and tissue conditioners [125]. Furthermore, additives like silver-quaternary ammonium silane (Ag-QAS) are incorporated into urethane dimethacrylate (UDMA) resins to counteract the pro-biofilm effect of biodegradation products like urethane methacrylate (UMA) [142].

*Advanced Functionalities*

**Figure 4:**
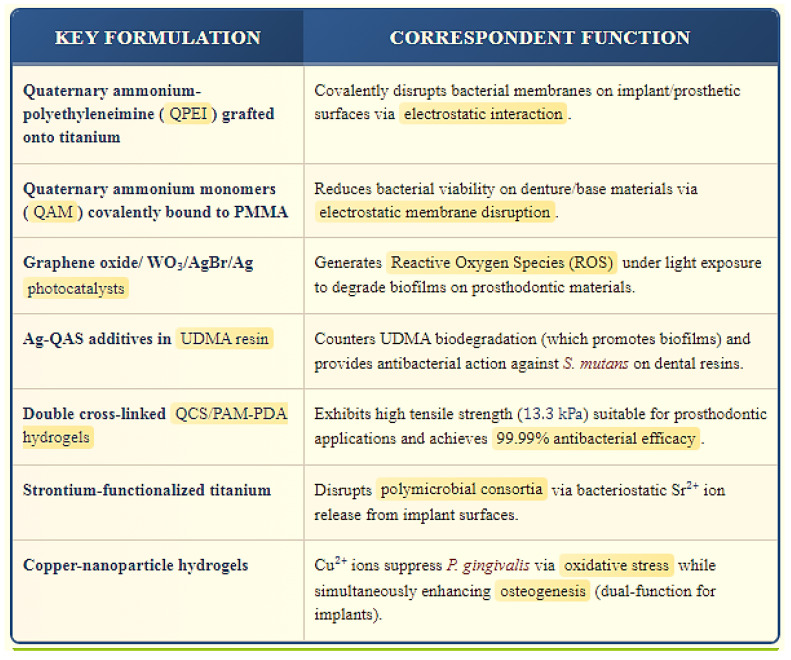
Top Emerging Smart Bioactive & Antibacterial Materials in Prosthodontics [136,137,138,139,140,141,142,143,144,145,146]. This selection was curated by authors based on criteria such as emerging innovations, possession of ‘smart’/advanced mechanisms (e.g., novel functionalities like covalent grafting, photocatalysis (ROS), pH/enzyme targeting, ion release, or dual strength/antibacterial action), and clear prosthodontic applicability.

Beyond core antimicrobial action, emerging smart materials exhibit intricate “smart” functionalities, often responding to environmental triggers. Sequential drug delivery is a key advancement, exemplified by pore-closed polylactic-co-glycolic acid (PLGA) microparticles embedded within chitosan hydrogels. This system provides an initial burst release of vancomycin to combat acute infection, followed by the sustained release of recombinant human bone morphogenetic protein-2 (rhBMP-2) to promote osteogenesis, important for bone-integrated prosthetics [128]. As investigated by Yu et al. in 2023, emerging smart materials like quaternary ammonium methacrylates (QAMs) and GO enable autonomous, pH-responsive caries prevention by selectively killing bacteria in acidic biofilm microenvironments, releasing remineralizing agents when most needed (low pH), and maintaining mechanical integrity despite acid challenges, with future advancements potentially integrating multi-stimuli-responsive systems (pH + enzyme + redox) for even smarter, precision-guided dental therapies [22]. Multifunctionality is a hallmark of many systems; copper-nanoparticle hydrogels not only suppress pathogens like *Porphyromonas gingivalis* via Cu^2+^-induced oxidative stress but also concurrently enhance osteogenic activity, addressing both infection control and tissue integration needs around implants [141,143,144]. Similarly, strontium-functionalized titanium provides bacteriostatic ion release while potentially offering osseointegration benefits inherent to strontium [145,146]. These integrated functionalities highlight the evolution from passive antimicrobial materials towards actively responsive, therapeutically intelligent platforms for advanced prosthodontics (Figure 4).

The landscape of antimicrobial strategies in prosthodontics is being transformed by smart materials like QACs, GO composites, and advanced hydrogels. By leveraging diverse mechanisms—from electrostatic membrane disruption and photocatalytic ROS generation to targeted ion release and enzyme-assisted degradation—and incorporating them into sophisticated formulations such as covalently modified surfaces, multifunctional nanocomposites, and stimuli-responsive delivery systems, these materials offer unprecedented capabilities. Their ability to provide not only potent, long-lasting antimicrobial action but also important secondary functions like mechanical reinforcement, osteoinduction, and programmed drug release positions them as essential tools for developing next-generation, infection-resistant prosthodontic devices and restorations.

## 4. Clinical Applications in Prosthodontics

**Figure 5:**
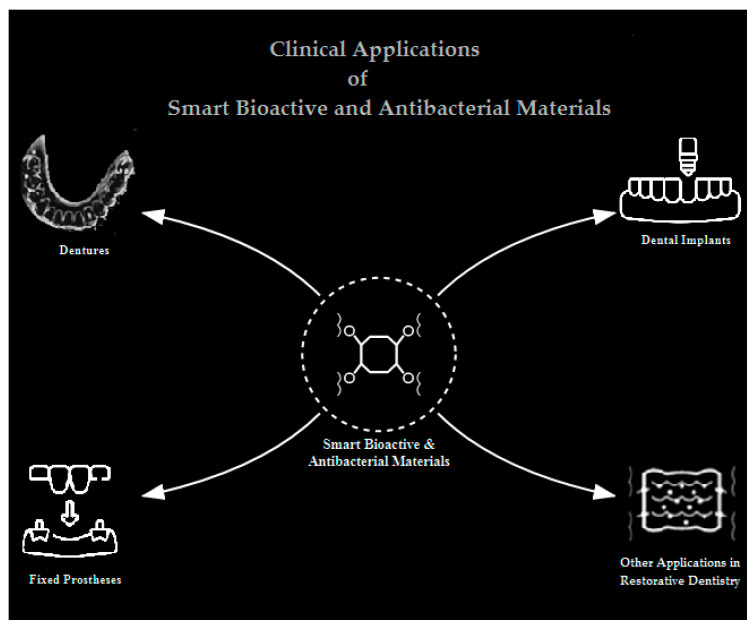
Applications of Smart Bioactive and Antibacterial Materials in Restorative Dentistry.

The integration of smart bioactive and antibacterial materials into prosthodontics has revolutionized infection control strategies, addressing biofilm-mediated complications such as denture stomatitis, peri-implantitis, and tooth decay. These innovations span dentures, dental implants, fixed prostheses, and other specialized applications, leveraging bioactive glass, nanoparticle-doped polymers, and pH-responsive coatings to enhance antimicrobial efficacy while maintaining mechanical and biological performance (Figure 5).

### 4.1. Dentures: Combating Candida Biofilms and Denture Stomatitis

Denture stomatitis, a prevalent inflammatory condition affecting denture wearers, is primarily driven by *Candida albicans* biofilm formation on acrylic surfaces [147,148]. The persistent colonization of fungal pathogens not only compromises oral health but also challenges the longevity of denture materials.

#### 4.1.1. Targeting Candida Biofilm Adhesion and Growth

A key focus in preventing denture stomatitis lies in disrupting *C. albicans* adhesion to polymethyl methacrylate (PMMA) surfaces. Silver nanoparticles (AgNPs) have emerged as a potent solution, with AgNP-doped acrylic resins effectively inhibiting fungal biofilm formation without sacrificing flexural strength [104,149]. Further enhancing this approach, β-AgVO_3_-doped denture adhesives demonstrate broad-spectrum efficacy, suppressing not only Candida but also multispecies oral biofilms [110,150]. For even greater antifungal precision, nystatin-coated AgNPs embedded in PMMA combine the mechanical benefits of acrylic with targeted drug delivery [151,152].

Beyond silver-based strategies, chitosan, a naturally derived biopolymer, offers a biocompatible alternative. Low-molecular-weight chitosan solutions (3–6 mg/mL) disrupt established *C. albicans* biofilms on PMMA, while also reducing adhesion of complex oral microcosms by over 75% [119,131,153]. When formulated into nanoparticles (CSNPs), chitosan directly degrades fungal biomass, and its versatility allows for synergistic combinations, such as CSNPs loaded with Mentha piperita essential oils to concurrently combat *Streptococcus mutans* [130,154]. High-molecular-weight chitosan gels provide an additional barrier, entirely preventing Candida adherence to acrylic resin surfaces [132,155].

#### 4.1.2. Sustained Antimicrobial Protection in Tissue Conditioners

Tissue conditioners, which cushion dentures and protect the oral mucosa, serve as an ideal medium for sustained antifungal delivery. Incorporating hybrid nanoparticles, such as Ag-ZnO-chitosan composites (2.5% *w*/*w*), reduces *C. albicans* colonization by 3–5 log CFU/mL, offering long-term fungal suppression [156,157]. These multifunctional systems leverage the combined antimicrobial properties of silver, zinc oxide, and chitosan, ensuring broad-spectrum efficacy while maintaining material integrity [158,159].

### 4.2. Dental Implants: Enhancing Osseointegration and Preventing Peri-Implantitis Through Advanced Surface Modifications

The long-term success of dental implants depends on two critical factors: the prevention of bacterial colonization and the promotion of osseointegration. Recent advancements in surface modifications have yielded coatings that not only provide robust antimicrobial protection but also actively enhance bone integration and soft-tissue healing. Among the most promising strategies are bioactive glass ceramics and nanoparticle-based coatings, which exhibit dual functionality by inhibiting pathogenic biofilms while fostering osteogenic activity.

#### 4.2.1. Surface Modifications for Antibacterial Efficacy

A diverse array of coatings provides persistent antimicrobial shields. Copper-doped mesoporous bioactive glass (MBG)/chitosan coatings inhibit *Porphyromonas gingivalis* (achieving a 5-fold reduction) while paradoxically accelerating endothelial cell migration, promoting soft tissue integration [61,160]. Silver-based strategies are prominent: Ag^+^-doped mesoporous bioactive glass films (e.g., 80SiO_2_-15CaO-5P_2_O_5_) on titanium inhibit *Aggregatibacter actinomycetemcomitans* (>80%) while inducing apatite nucleation for enhanced bioactivity [134,161,162]. AgNP-TiO_2_ nanotube coatings significantly reduce *Staphylococcus aureus* biofilm formation, with sequential application methods enabling sustained Ag^+^ release (4.05 ppm) for prolonged efficacy against *S. aureus* [111,163,164]. Ag-doped hydroxyapatite (Ag-HA) nanocoatings offer near-sterile implant surfaces [165,166]. Strontium-functionalized titanium (Sr-Ti-O) coatings disrupt polymicrobial consortia, specifically reducing *P. gingivalis* [146,167]. Zinc-chitosan/gelatin coatings applied via electrophoretic deposition mitigate bacterial attachment on abutments [62,168]. Chitosan-conjugated AgNPs (Ag-Chi) inhibit adhesion and quorum sensing in *S. mutans* and *P. gingivalis* on titanium without cytotoxicity [112,169]. Ion-implanted AgNPs reduce *S. aureus* colonization by 64.6% [117,170]. Antimicrobial peptide (AMP) coatings also impede *P. gingivalis* biofilm formation [171,172]. Quaternary ammonium compounds, like QPEI grafted onto titanium, disrupt bacterial membranes electrostatically [137,173].

#### 4.2.2. Enhancement of Osseointegration and Angiogenesis

Beyond their antibacterial properties, advanced coatings actively stimulate bone formation and vascularization. Copper-doped chitosan/BG coatings exhibit a dual mechanism, suppressing *P. gingivalis* colonization (fivefold reduction) while accelerating endothelial cell migration, thereby enhancing peri-implant vascularization. Silver-doped hydroxyapatite (Ag-HA) nanocoatings achieve near-sterile surfaces with a 97.5% reduction in biofilm formation, ensuring both antimicrobial protection and osteoconductivity. Zirconia-doped bioactive glass ceramic (Zr-BGC) coatings further promote rapid osseointegration, while zeolitic imidazolate frameworks (ZIF-8) loaded with naringin enable pH-responsive Zn^2+^ release, synergizing antibacterial and osteogenic effects.

#### 4.2.3. Peri-Implantitis Prophylaxis and Treatment

Smart materials offer novel approaches for managing peri-implant infections. Air abrasion using specific BG formulations (e.g., S53P4, Zn4) effectively eradicates biofilms (including *S. mutans*) from contaminated implant surfaces, with Zn^2+^-releasing BGCs specifically targeting periodontal pathogens. Non-surgical mechanical debridement is enhanced by oscillating chitosan brushes (OCB), which resolve mild peri-implantitis clinically, significantly reducing bleeding indices (73% reduction) and stabilizing radiographic bone levels over 12 months compared to conventional methods [21,174,175]. Photothermal strategies are emerging, such as simvastatin-loaded chitosan hydrogels activated by near-infrared (NIR) light to combat peri-implantitis via triggered drug release [176,177].

Collectively, these innovations underscore the transformative potential of multifunctional coatings in dental implantology. By integrating antibacterial efficacy with bioactive properties, modern surface modifications not only mitigate infection risks but also actively foster osseointegration and soft-tissue healing, ensuring long-term implant success.

### 4.3. Fixed Prostheses and Restorations

The longevity and success of fixed prostheses, such as crowns, bridges, and inlays/onlays, depend on their ability to resist secondary caries and bacterial colonization. Recent advancements in antibacterial materials and smart delivery systems have significantly enhanced the protective and functional properties of these restorations.

A key strategy involves the modification of glass ionomer cements (GICs) with fluoride-releasing chitosan nanoparticles, which reduce dentinal demineralization depth by 14% compared to conventional GICs, offering superior resistance to recurrent caries [122,178]. Beyond fluoride release, pH-responsive antimicrobial systems further bolster protection. For instance, mesoporous silica nanoparticles loaded with chlorhexidine and silver (Ag-MSNs@CHX) demonstrate dual antimicrobial action, suppressing *Streptococcus mutans* biofilms through pH-triggered release [179,180].

In CAD/CAM materials, antibacterial modifications have expanded the utility of both resins and ceramics. Resin-based materials incorporating e-poly-L-lysine and bioactive glass (F18) exhibit enhanced color stability and flexural strength while inhibiting biofilm formation [181]. Similarly, PMMA/Novaron (Ag/ZrPO_4_) and T-ZnO composites reduce bacterial adhesion by over 95% while improving mechanical durability [115,182]. Zirconia, a cornerstone of fixed prosthodontics, has been functionalized with chlorogenic acid-chitosan coatings, which not only inhibit *S. mutans* but also promote osteogenesis [127,183]. Additionally, TiO_2_/chitosan-modified zirconia demonstrates specific anti-adhesive effects against cariogenic bacteria.

Despite these advances, concerns remain regarding resin degradation by oral bacteria, such as UDMA hydrolysis by *S. mutans*. To counteract this, silver-quaternary ammonium salts (Ag-QAS) have been integrated into resin formulations, mitigating biodegradation while sustaining antimicrobial efficacy.

Together, these innovations, ranging from fluoride-releasing GICs to smart pH-responsive nanoparticles and antibacterial CAD/CAM materials, represent a breakthrough in fixed prosthodontics, ensuring restorations that are not only mechanically robust but also biologically resilient against the oral microbiome.

### 4.4. Other Restorative Applications: Expanding the Scope of Smart Materials

While Section 4.1, Section 4.2 and Section 4.3 focused on dentures, implants, and fixed prostheses, smart materials also play critical roles in niche restorative applications. This section details their use in bone graft substitutes, pH-responsive coatings, and guided bone regeneration (GBR), emphasizing mechanistic insights and clinical outcomes.

#### 4.4.1. Bone Graft Substitutes and Enhancers

Bioactive glass ceramics (BGCs) are increasingly used to fill peri-implant defects and augment bone regeneration due to their osteoconductive ion release (Ca^2+^, PO_4_^3−^, Si^4+^) and antimicrobial properties (Ag^+^, Zn^2+^, Sr^2+^, Cu^2+^) [44,58,59,60]. Main advancements include:

- Sr^2+^-functionalized coatings: Strontium-doped BGCs (e.g., Sr-Ti-O) inhibit *Porphyromonas gingivalis* while promoting osteoblast activity, achieving 3.5-fold higher angiogenesis vs. non-coated grafts [63,146].
- Cu-doped mesoporous BGCs (Cu-MBG): These grafts suppress pathogens (5-fold reduction in *P. gingivalis*) while accelerating endothelial cell migration, fundamental for vascularized bone repair [61,98].
- Bacteriostatic alkalinity: High pH from SiO_4_^4−^/Ca^2+^ release disrupts acidogenic biofilms (e.g., *Streptococcus mutans*), with Zn^2+^-BGCs showing >90% biofilm reduction in air-abrasion debridement [78,79,80].

Clinical Challenge: Dose-dependent cytotoxicity, e.g., Cu^2+^ ≥ 10 wt% impairing fibroblast proliferation [184].

#### 4.4.2. pH-Responsive Coatings for Targeted Therapy

These smart systems find specific niches exploiting the acidic microenvironment of biofilms. Chitosan (CS) and hybrid coatings leverage acidic biofilm microenvironments (pH < 5.5) for triggered drug release [123,124,125]:

1. Denture liners: CS-citral-Zn^2+^ complexes release Zn^2+^ at low pH, reducing *S. aureus* and *E. coli* adhesion by 90% [123].
2. Implant coatings:
  - Chitosan-gallium bilayers: Gallium release under acidity inhibits bacterial iron metabolism, synergizing with CS’s cationic disruption [126].
  - Aspirin/amoxicillin microparticles: pH-dependent erosion enables 99% metabolic biofilm reduction while enhancing osteogenesis (rBMSC adhesion) [134].
3. Sequential delivery systems: Pore-closed PLGA microparticles in CS hydrogels deliver vancomycin (burst) followed by rhBMP-2 (sustained), combining infection control and osseointegration [128].

Limitation: Excessive protonation at low pH may destabilize coatings.

#### 4.4.3. Guided Bone Regeneration (GBR) Membranes

Smart GBR membranes integrate antimicrobial and regenerative functions:

- CuNP-chitosan hydrogels: Cu^2+^ suppresses *S. aureus* and *P. gingivalis* via oxidative stress while supporting patient-specific GBR [141].
- AgNP-coated collagen: Reduces infection risk in GBR procedures, with 97.5% biofilm inhibition on titanium surfaces [165].
- 4D-printed hydrogels: Simvastatin-loaded ZIF-8@PDA systems enable photothermal drug activation, targeting peri-implantitis [176,177].

Future Direction: AI-driven design of multifunctional membranes (e.g., QAC + BGC + CS) may optimize antimicrobial-mechanical balance [22].

## 5. Discussion. Navigating Challenges and Charting Future Trajectories for Smart Bioactive Materials in Restorative Dentistry

The burgeoning field of smart bioactive and antibacterial materials heralds a transformative era in dentistry, promising devices capable of autonomous infection control and enhanced tissue integration. However, the path from promising laboratory innovation to widespread clinical adoption is fraught with significant scientific, translational, and regulatory complexities, demanding nuanced solutions and concerted research efforts.

### 5.1. Challenges Impeding Clinical Translation

The clinical realization of smart bioactive materials faces a multi-faceted array of hurdles, with toxicity concerns representing a paramount challenge, particularly for materials leveraging potent antimicrobial metal ions like Ag^+^ or Cu^2+^. As Ben-Arfa et al. have demonstrated, high Cu^2+^ loading (≥10 wt%) can significantly inhibit fibroblast proliferation, a critical process for soft tissue healing around prostheses [184]. Similarly, AgNPs, while highly effective against pathogens, present a delicate balancing act. Gunputh et al., Pokrowiecki et al., Sun et al., and Jiang et al. collectively highlight that concentrations exceeding 0.1–0.5 ppm in solution or >0.3 wt% incorporated into PMMA can trigger fibroblast apoptosis via caspase-3 activation or induce osteoblast cytotoxicity [111,185,186,187]. Furthermore, Guo et al. corroborate that such elevated AgNP concentrations impair fibroblast proliferation [31]. This cytotoxicity is often mechanistically linked to uncontrolled ion leaching and subsequent cellular damage, necessitating precision-engineered controlled release strategies such as nHA-capping, confinement within UiO-66 metal–organic frameworks (MOFs), or sophisticated bilayer systems. Compounding this issue, as observed with AgVO_3_, is the concerning emergence of pathogen resistance, exemplified by intrinsic resistance mechanisms in *C. albicans* biofilms involving extracellular polymeric substance (EPS) shielding and efflux pump upregulation to evade Ag^+^ toxicity, highlighting the need for multifunctional antimicrobial designs.

BGCs deliver therapeutic ions (Ca^2+^, PO_4_^3−^, Zn^2+^, Sr^2+^), but their clinical promise is tempered by dose-dependent toxicity. High Ag^+^ or Cu^2+^ concentrations induce fibroblast and osteoblast cytotoxicity via reactive oxygen species (ROS) generation, while excessive alkaline ion release (e.g., Ca^2+^, SiO_4_^4−^) elevates local pH, risking tissue necrosis [188,189]. Furthermore, silica-rich BGCs may provoke low-grade inflammation through macrophage activation, and nanoparticle aggregation can exacerbate immunogenic responses [190]. Mitigation approaches, such as Sr^2+^ substitution for Ag^+^ or polymer-BGC hybrid designs, aim to refine ion release kinetics while preserving bioactivity.

**Figure 6:**
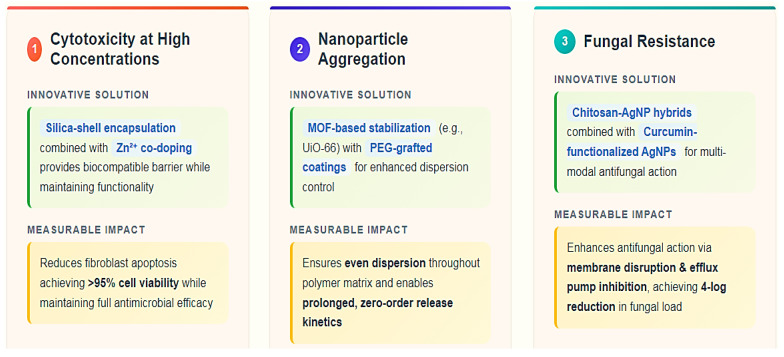
Key Challenges and Material-Specific Mitigation Strategies for AgNP-Doped Polymers.

AgNP-polymers represent a paradigm shift in antimicrobial prosthodontics, yet their clinical adoption is hindered by three fundamental challenges rooted in nanomaterial behavior and biological interactions:

- Cytotoxicity at high concentrations: Concentrations beyond 0.3 wt% in PMMA compromise fibroblast viability, necessitating diffusion-barrier strategies like silica-shell encapsulation or Zn^2+^ co-doping to suppress pro-apoptotic pathways.
- Nanoparticle aggregation: Uncontrolled agglomeration reduces antimicrobial efficacy and increases localized toxicity. Advanced stabilization methods, such as UiO-66 MOF confinement or PEG-based steric shielding, are critical to maintaining colloidal stability.
- Fungal resistance: *C. albicans* adapts via EPS production and efflux mechanisms, prompting the development of synergistic agents like chitosan-AgNP hybrids or curcumin-functionalized AgNPs to disrupt biofilm defenses (Figure 6).

Chitosan’s cationic nature underpins its antimicrobial activity but also introduces pH-sensitive toxicity risks. In acidic environments (e.g., carious lesions), excessive protonation disrupts host cell membranes through electrostatic interactions, while residual chitin-derived impurities may trigger allergic reactions. Burst release of co-delivered antimicrobials (e.g., Ag^+^, Zn^2+^) risks dysbiosis or resistance, and swelling-induced delamination raises safety concerns. Crosslinking with genipin or glutaraldehyde and rigorous purification are key strategies to enhance coating stability and biocompatibility [191].

The path to clinical adoption of smart bioactive materials demands rigorous toxicity management. While BGCs require controlled ion release to avoid pH shifts and oxidative damage, AgNP-polymers need nanoengineering to prevent aggregation and resistance. Chitosan coatings, though versatile, must address charge-dependent cytotoxicity (Table 3).

Additionaly, the field grapples with substantial clinical translation and regulatory barriers. A critical deficiency, emphasized by Wareham-Mathiassen et al. and Bento de Carvalho et al., is the lack of standardized, internationally recognized protocols (e.g., ISO standards) for rigorously evaluating antibacterial durability and biofilm-disruption efficacy under clinically relevant conditions, such as mandating a ≥3-log CFU/mL reduction [192,193]. Standardized in vivo protocols capable of concurrently assessing biofilm inhibition kinetics and osseointegration outcomes are urgently needed, especially given the complex interplay where material biodegradation might paradoxically fuel biofilm growth. Importantly, as noted by the scientific community, there exists a stark paucity of long-term human clinical data for many highly promising materials, including Nb_2_O_5_-modified bioactive glasses, Zn-core nanoparticles, and Sr-Ti-O coatings. Navigating the regulatory hurdles and commercialization pathways for these novel materials remains daunting, encompassing the complex safety assessment of nanoparticles and the regulatory classification of innovative substances like CBD-infused PMMA [194]. Palaskar et al. also highlight the challenge of ensuring long-term stability, preventing nanoparticle leaching or uncontrolled ion release that could compromise biocompatibility or material integrity over the prosthesis lifespan [195].

### 5.2. Envisioning Future Directions: Innovation and Standardization

The horizon for smart prosthodontic materials is illuminated by the revolutionary potential of 3D/4D printing for customized smart prostheses. This advanced manufacturing paradigm, championed by researchers like Liu et al. (2025), enables the fabrication of patient-specific devices with unprecedented spatial control over bioactivity and complex, dynamic drug delivery profiles [177]. Exemplary systems under investigation include 4D-printed Sim@ZIF8@PDA hydrogels enabling on-demand photothermal/immunomodulatory therapy, self-healing chitosan hydrogels adaptable to individual anatomy, and CuNP hydrogels designed for guided bone regeneration membranes [141,196,197]. The sophistication achievable is further highlighted by sequential drug delivery platforms, such as those providing an initial vancomycin burst to combat acute infection followed by sustained release of rhBMP-2 to foster osteointegration [128].

A summary of the most promising materials points to several standout candidates, as identified by the reserach community. Bioactive glass ceramics (S53P4, F18, Ag^+^ doped variants, Zn-BGs) remain highly attractive due to their dual capacity for osseoconductive ion release and antimicrobial action. Chitosan-based systems, particularly pH-responsive formulations, offer targeted drug delivery and membrane disruption specifically within acidic biofilm microenvironments. Engineered silver nanoparticles (β-AgVO_3_, Ag-Chi hybrids, Zn-core/C-shell structures) integrated into controlled-release polymeric matrices continue to show exceptional promise when cytotoxicity is managed. Specific advanced formulations demonstrating the critical balance between potent antibiofilm efficacy (>90–99% reduction) and biocompatibility include pH-responsive chitosan-gallium bilayers, AgNP-doped SiO_2_ nanocomposites, Ag-HA nanocoatings, and quaternary ammonium chitosan (QAC) hydrogels. The integration of stimuli-responsive mechanisms (pH, redox, light) is important for enabling precise, on-demand therapeutic action at the infection site. Multifunctional hybrid materials, such as combinations of bioactive glass ceramics (BGC), quaternary ammonium compounds (QAC), and chitosan (CS), are emerging for their potential synergistic effects.

Underpinning all future progress is the imperative for a call for standardized clinical testing protocols [198]. These protocols need to mandate quantifiable metrics for biofilm-disruption (e.g., ≥3-log CFU/mL reduction) and incorporate comprehensive long-term (e.g., 12-month minimum) clinical tracking to evaluate both sustained antimicrobial efficacy and functional outcomes like osseointegration concurrently [199]. Developing robust in vitro-in vivo correlation (IVIVC) models, as advocated by Yu et al., Hu et al., Chai et al., and Shi et al., is needed to reliably bridge the gap between promising bench-top results and predictable clinical performance [109,134,176,200].

### 5.3. Future Research

**Figure 7:**
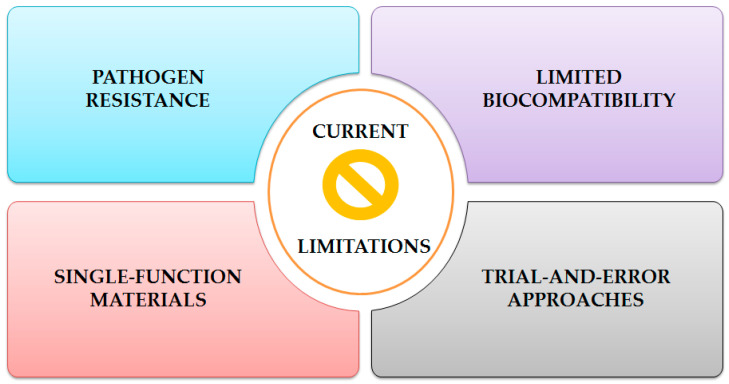
Limitations faced by current antimicrobial materials development.

Contemporary antimicrobial material development faces multifaceted challenges, including emerging pathogen resistance mechanisms, restricted biocompatibility profiles that limit clinical applications, predominantly single-function material designs that fail to address complex therapeutic requirements, and inefficient trial-and-error methodologies that impede systematic optimization of material properties (Figure 7).

**Figure 8:**
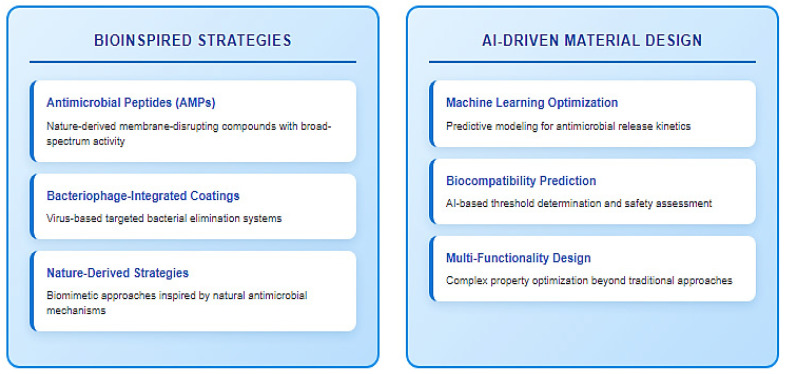
Solutions for future antimicrobial materials development.

The exploration and development of bioinspired materials offer exciting avenues to overcome limitations like pathogen resistance. Liu et al. (2025) highlight the promise of antimicrobial peptides (AMPs), bacteriophage-integrated coatings, and other nature-derived strategies [171]. Concurrently, machine learning-driven material design and broader AI-driven material design are poised to revolutionize the field, enabling the optimization of complex properties such as antimicrobial release kinetics, biocompatibility thresholds, and multi-functionality far more efficiently than traditional trial-and-error approaches (Figure 8).

**Figure 9:**
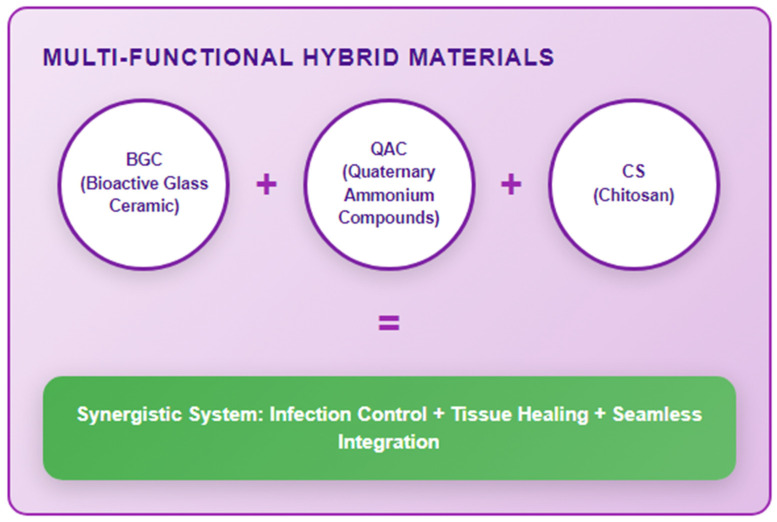
Future design of multi-functional hybrid materials.

The design of multi-functional hybrid materials (e.g., BGC + QAC + CS combinations) aims to harness synergistic effects, creating systems that simultaneously combat infection, promote tissue healing, and integrate seamlessly (Figure 9).

Ultimately, only through comprehensive long-term clinical trials to validate biocompatibility and efficacy can these advanced materials gain the necessary trust for widespread clinical adoption.

### 5.4. Limitations and Strenghts of This Review

The preliminary research led to the authors’ choice to establish the scope of the review on innovations in Bioactive Glass, AgNP Polymers, and CS-based pH-Responsive Coatings in restorative dentistry. As such, this review explicitly states its limitations based on its focus on restorative dentistry and the selection of some of the most relevant smart bioactive and antibacterial materials for restorative dentistry, acknowledging that smart materials encompass a broad subject matter with applications across various industries. Moreover, this review does not aim to exhaustively detail every smart material or every aspect of this subject.

While this discussion synthesizes key challenges and opportunities, it is constrained by the inherent vastness and rapid evolution of the field of smart bioactive and antibacterial materials for restorative dentistry. The sheer diversity of novel formulations, nanoparticles, composite strategies, and emerging mechanisms (e.g., photodynamic, immunomodulatory) makes an exhaustive coverage of every promising avenue impractical within a single review. Furthermore, the relative newness of many advanced platforms, particularly those leveraging 4D printing or complex AI-designed hybrid materials, means long-term clinical data and real-world performance validation are still maturing. Consequently, this analysis primarily reflects the current state of knowledge and prominent research trajectories, acknowledging that the landscape continues to shift rapidly with ongoing innovation.

Nevertheless, this review is among the first to consolidate advancements in smart bioactive materials (BGCs, AgNP polymers, CS coatings) specifically for restorative dentistry, with a focus on stimuli-responsive antibiofilm and tissue-regenerative functionalities. While prior reviews have addressed individual material classes, this work uniquely:

- Integrates pH/enzyme-responsive mechanisms with clinical outcomes (e.g., Section 4 on denture stomatitis and peri-implantitis).
- Critically evaluates translational challenges (e.g., Table 3 on cytotoxicity thresholds) and proposes standardized protocols.
- Highlights innovations like 4D-printed hydrogels and AI-driven design (Section 5.2), not previously reviewed in this context.

This interdisciplinary approach bridges gaps between material science, microbiology and clinical dentistry, offering actionable insights for future research.

## 6. Conclusions

While AgNP-polymers provide the highest antibacterial potency for passive devices and chitosan coatings offer the most intelligent, targeted delivery, Bioactive Glass Ceramics (BGCs) represent the most transformative advancement for restorative dentistry overall. Their unique ability to provide robust, broad-spectrum antimicrobial action while actively promoting tissue regeneration and osseointegration fulfills the core clinical needs in implantology and bone repair, making them the foundation upon which next-generation, truly bioactive restorations will be built.

Future progress hinges on developing these materials into controlled-release hybrid systems and validating their performance in long-term clinical trials with standardized metrics.

## Figures and Tables

**Table 1:** Biological Roles and Clinical Impact of Key Therapeutic Ions in Engineered BGCs [63,89,90,91,92,93,94,95,96].

Ion	Primary Biological Target	Mechanism of Action	Clinical Impact
**Cu^2+^**	Angiogenesis & Osteogenesis	- VEGF secretion (promotes blood vessel formation); - RUNX2/Osteocalcin (osteogenic markers).	Accelerates vascularization and osseointegration of implants; improves bone-implant healing.
**Zn^2+^**	Antimicrobial & Osteo-support	- Membrane disruption in microbes; - ROS (antimicrobial); - Enzyme co-factor (e.g., ALP for bone formation).	Reduces peri-implantitis risk; supports bone metabolism and mineralization.
**Sr^2+^**	Osteoblast/Osteoclast Balance	- Osteoblast activity (bone formation); - Osteoclast resorption (bone breakdown).	Enhances bone density around implants; useful in osteoporotic patients.
**Ag^+^**	Broad-Spectrum Antimicrobial	- Membrane disruption; - Protein/DNA denaturation in pathogens.	Prevents bacterial/fungal infections; reduces biofilm formation on implants.

**Table 2:** Key Chitosan-Based pH-Responsive Formulations for Antimicrobial and Drug Delivery Applications in Dentistry [62,87,118,119,120,121,122,123,124,125,126,127,128,129,130,131,132,133,134,135].

Key Formulation	Correspondent Function
**Chitosan-Citral Schiff Base (Zn^2+^ loaded)**	Releases Zn^2+^ in acidic biofilm microenvironments; Antibacterial effects against *S. aureus*, *E. coli*, oral pathogens
**Fluoride-loaded Chitosan Nanoparticles in GIC**	Reduces secondary caries depth by 9%; Inhibits *S. mutans*; Enhances remineralization
**Chitosan-based pH-responsive Coating (General)**	Exhibits cationic disruption of microbial membranes; Controlled drug release under acidic biofilm conditions
**Aspirin/Amoxicillin co-loaded Microparticles**	pH-dependent erosion enables sustained dual-drug elution; Achieves 99% metabolic biofilm reduction; Enhances rBMSC adhesion
**Zn^2+^-chelated Chitosan/Gelatin**	Disrupts quorum sensing and bacterial adhesion (90% reduction); Accelerates human gingival fibroblast migration
**Chitosan Nanoparticles (30.1 μg/mL)**	Cationic disruption of fungal membranes; Reduces *Candida* biomass by 51.5% on acrylic resin
**Low-MW Chitosan Solutions (3–6 mg/mL)**	Disrupts *C. albicans* biofilms on PMMA resin through electrostatic membrane destabilization
**Chitosan/Ethylcellulose Nanocomposite**	Enables redox/pH-triggered drug release
**Protonated Chitosan Amines (Acidic pH)**	Enhances biofilm penetration and drug release (e.g., gallium, D-arginine)
**High-MW Chitosan Gel**	Inhibits 100% *C. albicans* adherence to acrylic resin (*p* < 0.001)
**Lysostaphin-Chitosan Hydrogel**	Reduces *S. aureus* viability by ∼3 Log_10_ CFU/mL ex vivo
**Quaternary Ammonium Chitosan/Alginate LBL Coatings**	Reduces *Candida* biofilms > 90% post-mechanical stress
**Pore-closed PLGA MPs in Chitosan Hydrogel**	Enables sequential vancomycin (burst) and rhBMP-2 (sustained) release
**ZIF-8 (Naringin loaded) on TiO_2_ Nanotubes**	Enables pH-responsive Zn^2+^ release; Synergizes antibacterial and osteogenic effects

**Table 3:** Paths toward Clinically Viable Solutions.

Material	Primary Toxicity Mechanism	Critical Threshold	Key Mitigation Strategy
**BGCs**	Ag^+^/Cu^2+^-induced ROS, pH imbalance	≥10 wt% Cu^2+^ inhibits fibroblasts	Sr^2+^ substitution, polymer hybridization
**AgNP-Polymers**	Ag^+^ leaching, nanoparticle aggregation	>0.3 wt% in PMMA	MOF confinement, silica-shell encapsulation
**Chitosan Coatings**	pH-dependent membrane disruption	High cationic density at low pH	Crosslinking, buffering agents

## Data Availability

Not applicable.

## Abbreviations

The following abbreviations are used in this manuscript:

PMMA	Polymethyl methacrylate
Ag^+^	Silver ion
Zn^2+^	Zinc ion
Sr^2+^	Strontium ion
BG	Bioactive glass
BGCs	Bioactive glass ceramics
S53P4	A specific bioactive glass composition (53% SiO_2_, 22% Na_2_O, 21% CaO, 4% P_2_O_5_)
F18	A bioactive glass formulation
HCA	Hydroxycarbonate apatite
RUNX2	Runt-related transcription factor 2 (osteogenic marker)
VEGF	Vascular endothelial growth factor
Zr-BGCs	Zirconium-modified bioactive glass ceramics
Nb_2_O_5_	Niobium pentoxide
Cu-MBG	Copper-doped mesoporous bioactive glass
AgNPs	Silver nanoparticles
ROS	Reactive oxygen species
β-AgVO_3_	Silver vanadate (antimicrobial agent)
UiO-66	A zirconium-based metal–organic framework (MOF)
nHA	Nano-hydroxyapatite
Ag-Chi	Chitosan-conjugated silver nanoparticles
CS	Chitosan
GIC	Glass ionomer cement
CSNPs	Chitosan nanoparticles
PLGA	Poly(lactic-co-glycolic acid)
rhBMP-2	Recombinant human bone morphogenetic protein-2
QAM	Quaternary ammonium monomers
QACs	Quaternary ammonium compounds
QPEI	Quaternary ammonium-polyethyleneimine
GO	Graphene oxide
ZIF-8	Zeolitic imidazolate framework-8
TiO_2_	Titanium dioxide
MoSe_2_	Molybdenum diselenide
UDMA	Urethane dimethacrylate
Ag-QAS	Silver-quaternary ammonium silane
LBL	Layer-by-layer (coating technique)
CFU	Colony-forming unit
MBG	Mesoporous bioactive glass
Ag-HA	Silver-doped hydroxyapatite
LSTC	Lysostaphin-chitosan hydrogel
CBD	Cannabidiol (in experimental PMMA formulations)
IVIVC	In vitro-in vivo correlation
